# Hierarchical identification of a transcriptional panel for the histological diagnosis of lung neuroendocrine tumors

**DOI:** 10.3389/fgene.2022.944167

**Published:** 2022-08-29

**Authors:** Juxuan Zhang, Jiaxing Deng, Xiao Feng, Yilong Tan, Xin Li, Yixin Liu, Mengyue Li, Haitao Qi, Lefan Tang, Qingwei Meng, Haidan Yan, Lishuang Qi

**Affiliations:** ^1^ College of Bioinformatics Science and Technology, Harbin Medical University, Harbin, China; ^2^ Department of Medical Oncology, Harbin Medical University Cancer Hospital, Harbin, China; ^3^ Basic Medicine College, Harbin Medical University, Harbin, China; ^4^ Department of Bioinformatics, Fujian Key Laboratory of Medical Bioinformatics, School of Medical Technology and Engineering, Fujian Medical University, Fuzhou, China

**Keywords:** relative gene expression orderings, transcriptional signatures, individualization, histological classification, lung neuroendocrine tumors

## Abstract

**Background:** Lung cancer is a complex disease composed of neuroendocrine (NE) and non-NE tumors. Accurate diagnosis of lung cancer is essential in guiding therapeutic management. Several transcriptional signatures have been reported to distinguish between adenocarcinoma (ADC) and squamous cell carcinoma (SCC) belonging to non-NE tumors. This study aims to identify a transcriptional panel that could distinguish the histological subtypes of NE tumors to complement the morphology-based classification of an individual.

**Methods:** A public dataset with NE subtypes, including 21 small-cell lung cancer (SCLC), 56 large-cell NE carcinomas (LCNECs), and 24 carcinoids (CARCIs), and non-NE subtypes, including 85 ADC and 61 SCC, was used as a training set. In the training set, consensus clustering was first used to filter out the samples whose expression patterns disagreed with their histological subtypes. Then, a rank-based method was proposed to develop a panel of transcriptional signatures for determining the NE subtype for an individual, based on the within-sample relative gene expression orderings of gene pairs. Twenty-three public datasets with a total of 3,454 samples, which were derived from fresh-frozen, formalin-fixed paraffin-embedded, biopsies, and single cells, were used for validation. Clinical feasibility was tested in 10 SCLC biopsy specimens collected from cancer hospitals via bronchoscopy.

**Results:** The NEsubtype-panel was composed of three signatures that could distinguish NE from non-NE, CARCI from non-CARCI, and SCLC from LCNEC step by step and ultimately determine the histological subtype for each NE sample. The three signatures achieved high average concordance rates with 97.31%, 98.11%, and 90.63%, respectively, in the 23 public validation datasets. It is worth noting that the 10 clinic-derived SCLC samples diagnosed via immunohistochemical staining were also accurately predicted by the NEsubtype-panel. Furthermore, the subtype-specific gene expression patterns and survival analyses provided evidence for the rationality of the reclassification by the NEsubtype-panel.

**Conclusion:** The rank-based NEsubtype-panel could accurately distinguish lung NE from non-NE tumors and determine NE subtypes even in clinically challenging samples (such as biopsy). The panel together with our previously reported signature (*KRT5*-*AGR2*) for SCC and ADC would be an auxiliary test for the histological diagnosis of lung cancer.

## 1 Introduction

Lung cancer is the most common malignant tumor and one of the main causes of cancer-related deaths in humans. The most common histological classification of lung cancer is small-cell lung cancer (SCLC) and non-small cell lung cancer (NSCLC), which is based on cell morphology, according to the World Health Organization (WHO) criteria. In 2015, the WHO updated this classification by providing a new criterion that classifies lung cancer into neuroendocrine (NE) and non-NE tumors based on NE morphology (Rekhtman, 2010; Travis et al., 2015), to provide new insights into precision therapy for lung cancer (Yang and Lin, 2016).

Lung NE tumors account for approximately 25% of all lung tumors and include SCLC (∼20%), large cell neuroendocrine carcinomas (LCNECs, ∼3%), and carcinoids (CARCIs, ∼2%) (Rekhtman, 2022). The treatment strategies for lung NE are different from those for non-NE and even differ for each NE subtype. The main treatment for SCLC is combination chemotherapy, typically with etoposide plus either cisplatin or carboplatin (Ramirez et al., 2021), while surgery is only performed on a few early-stage patients; this is different from the treatment modalities of other NE subtypes and non-NE patients (Lindeman et al., 2013). Chemotherapy schedule for LCNEC after surgical resection is typically adopting NSCLC or SCLC chemotherapy regimens, and this has always been controversial (Fasano et al., 2015). As per recent studies, etoposide–cisplatin chemotherapies, that is, “treat as an SCLC,” are more effective strategies for LCNEC patients (Fasano et al., 2015; Ramirez et al., 2021). For CARCI treatment (an NE subtype with low malignancy), the main therapy is surgical resection (Ramirez et al., 2021). These discrepancies in tumor biology and in response to drug treatment highlight the importance of distinguishing lung NE from non-NE tumors and determining the NE subtypes accurately.

Microscopic morphological features observed using hematoxylin–eosin (HE)-stained specimens are the “gold standard” for elucidating lung cancer histological classification. NE tumors have some unique morphological characteristics (organ-like structure, palisade or trabecular arrangement, and chrysanthemum-shaped cluster structure) and ultra-microstructures (dense core particles) (Teng et al., 2016), which can be used to distinguish them from non-NE tumors. For the NE subtypes, CARCI can be distinguished from SCLC and LCNEC based on the mitotic phases and necrosis degree; LCNEC, large cells with abundant cytoplasm and prominent nucleoli, can be distinguished from SCLC (small cells with sparse cytoplasm and inconspicuous nucleoli) based on cell morphological characteristics (Lantuejoul et al., 2020). However, all these diagnostic criteria have been described from surgical specimens, which can be difficult to demonstrate on small biopsy specimens (Hung, 2019), that they account for approximately 70% of the initial lung cancer diagnoses (Travis et al., 2013). As a result, a proportion of LCNEC tumors were recognized as large-cell carcinoma (LCC) on biopsy and cytology and subsequently misclassified as non-NE.

Therefore, immunohistochemical (IHC) detection of subtype-specific markers has been proposed for assisting histological classification. NE markers, such as chromogranin (CgA), synaptophysin (Syp), and CD56, can be used as auxiliary diagnostic tools for discriminating NE from non-NE tumors (Rekhtman, 2022). However, the classification accuracy of NE markers is limited by their suboptimal sensitivity and specificity (Teng et al., 2016; Rekhtman, 2022), because approximately 5–10% of NE tumors can be negative for all the above three NE markers (Yatabe et al., 2019). Several studies revealed that the diagnostic accuracies of the three NE markers (CgA, Syp, and CD56) were approximately 42, 40, and 88%, respectively (Park et al., 2003; Zhou et al., 2013). In addition, 10–20% of NSCLC without morphological features of NE neoplasms, which have similar cytological features to LCNEC, may also show expression of NE markers on IHC detection (Lantuejoul et al., 2020), leading to non-NE patients misdiagnosed as LCNEC.

It is important that even with the auxiliary immunomarkers, there is still a certain percentage of misclassified cases because of the subjective diagnoses of HE staining or immunostaining results made by pathologists using varying pathological criteria or interpretations, resulting in low reproducibility of pathological diagnosis between LCNEC and SCLC in particular (Thunnissen et al., 2017). Two previous studies have reported that there was a percentage of SCLC and LCNEC samples for which no consensus diagnosis could be reached among most pathologists (den Bakker et al., 2010; Ha et al., 2012). Moreover, some SCLC and LCNEC borderline subgroups with comparable features make accurate diagnosis challenging (Thunnissen et al., 2017; Sonkin et al., 2019). Furthermore, clinical pathological specimens, often derived from small biopsies, inevitably suffer from mechanical damage and squeezing, which typically lack a well-preserved morphology in most cases, rendering morphological and IHC evaluation difficult (Baine and Rekhtman, 2020).

Therefore, considerable efforts have been devoted to extracting signatures based on gene expression profiles to stratify the histological subtypes of lung cancer (Girard et al., 2016). However, most transcriptional signatures were developed to distinguish between adenocarcinoma (ADC) and squamous cell carcinoma (SCC) belonging to non-NE tumors (Girard et al., 2016; Li et al., 2019), and only a few studies focused on lung NE tumors. Faruki et al. developed a lung subtyping panel consisting of 57 genes for the diagnosis of ADC, SCC, and NE (Faruki et al., 2016), while it could not determine the NE subtypes. Guo et al. constructed a classifier based on transcriptome data to improve the diagnostic accuracy for LCNEC and SCLC (Guo et al., 2021). However, most of these reported quantitative transcriptional signatures lack robustness for clinical applications because of batch effects (Guan et al., 2018) and quality uncertainties of clinical samples, such as in formalin-fixed paraffin-embedded (FFPE) tissues with high RNA degradation and small biopsy specimens with low-input RNA (Chen et al., 2017; Liu et al., 2017). In contrast, the “within-sample” relative expression orderings (REOs) of gene pairs, which are the qualitative transcriptional characteristics of samples, are highly robust against experimental batch effects (Zheng et al., 2021; Li et al., 2022; Wang et al., 2022; Wu et al., 2022), partial RNA degradation during specimen storage and preparation (Chen et al., 2017), and low-input RNA specimens (Liu et al., 2017) and can be directly applied to samples at individualized levels (Qi et al., 2016). Before, we had developed a robust qualitative signature (*KRT5* and *AGR2*) for distinguishing SCC and ADC (non-SCC) subtypes based on the REO approach (Li et al., 2019). However, this signature invariably classifies lung cancer into SCC or ADC (non-SCC) categories; therefore, it is worthwhile to develop a panel of signatures based on REOs that can be used in a diagnostic context for all clinically important histological subtypes of lung cancer.

This study aimed to develop a panel of qualitative signatures step by step for distinguishing NE from non-NE tumors and determining NE subtypes individually. In the training dataset, consensus clustering was performed to exclude dubious samples whose expression patterns were discordant with their pathological subtypes and a rank-based method was applied to construct a panel of qualitative transcriptional signatures for the NE subtypes. The performance of the signatures was tested in independent datasets with multiple tissue types, even for the clinical challenging tissues (biopsies specimens). A tentative clinical cohort of 10 SCLC samples was collected to test the clinical feasibility. Gene expression patterns of the specific immunomarker genes and survival analyses were also conducted to support the reclassification obtained by the NEsubtype-panel.

## 2 Materials and methods

### 2.1 Public data sources and data preprocessing

**FIGURE 1:**
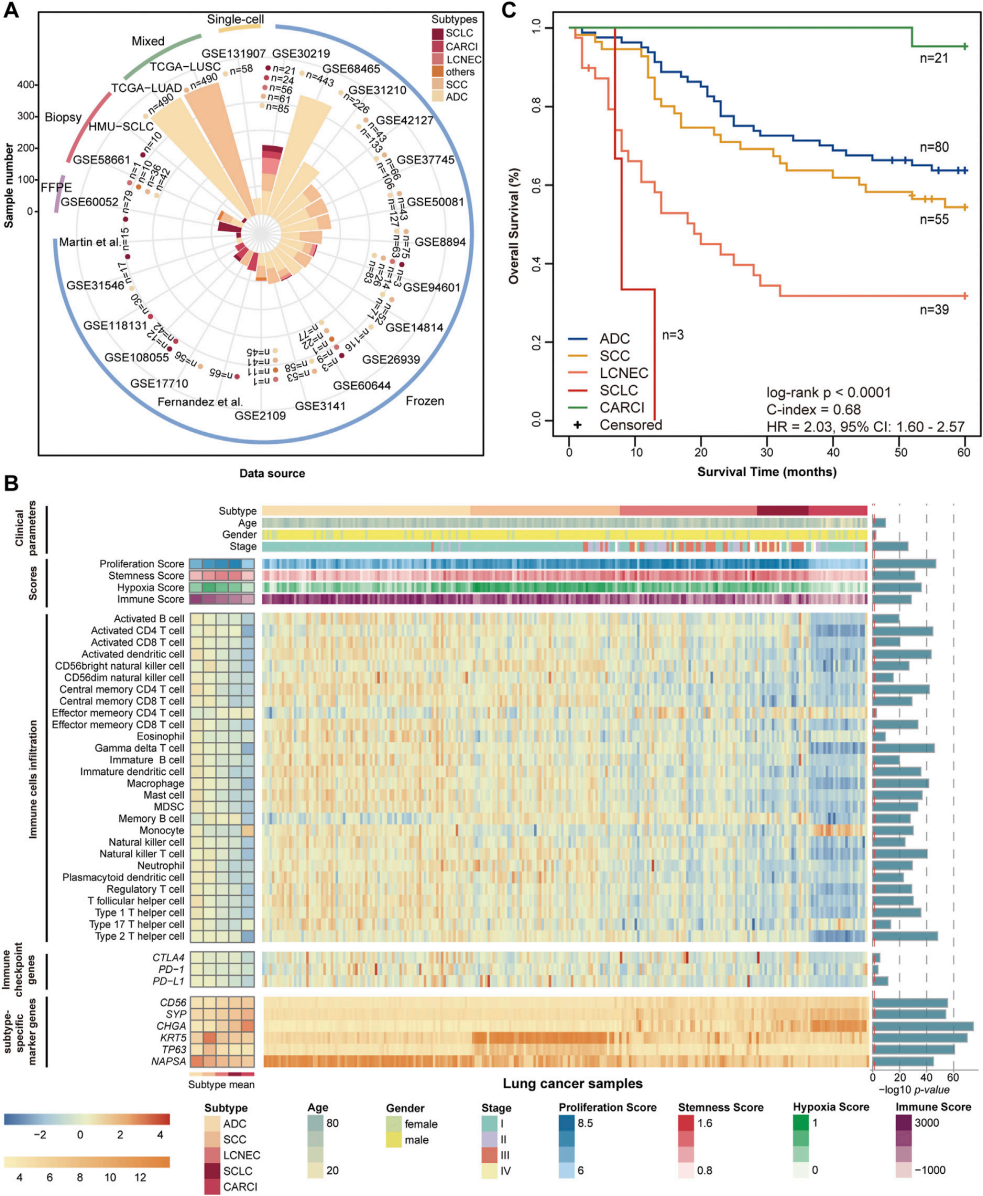
Datasets and molecular landscape of lung cancer. **(A)** 25 lung cancer datasets were used in this study. **(B)** heatmaps of the molecular landscape of lung cancer subtype in the training (GSE30219) dataset. The clinical heatmap panels show the distributions of clinical parameters, including histological subtype, tumor stage, age, and sex. The score heatmap panels show the proliferation scores, stemness scores, hypoxia scores, and immune scores calculated by mRNA expression profiles, based on the published articles (Supplementary Material). The boxplots of four scores across the lung cancer subtypes are displayed in Supplementary Figure S1. The immune cell heatmap panels show the relative infiltration abundances of 28 immune cell types quantified by ssGSEA. The immune checkpoint heatmap panels show the mRNA expression levels of three immune checkpoint genes, which are targets of immunotherapy. The levels of immune cell infiltration and immune checkpoint gene expression were scaled across all samples using the Z-score method. The subtype-specific marker heatmap panels depict the mRNA expression levels of seven subtype-specific marker genes, including three neuroendocrine marker genes (*CD56*, *SYP*, and *CHGA*), two SCC marker genes (*KRT5* and *TP63*), and one ADC marker gene (*NAPSA*). Analysis of variance was used to test the differences across five subtype groups. The log 10-transformed *p* values are displayed on the left of the heatmap panels. **(C)** Kaplan–Meier curves of overall survival for the lung cancer subtypes in the training (GSE30219) dataset. The patients had undergone only curative surgical resection. ssGSEA, single-sample gene set enrichment analysis; SCC, squamous cell carcinoma; and ADC, adenocarcinoma.

The 22 public gene expression datasets of lung tissues used in this study were downloaded from the Gene Expression Omnibus (GEO, http://www.ncbi.nlm.nih.gov/geo/) and The Cancer Genome Atlas (TCGA, http://cancergenome.nih.gov/). Two datasets were collected through a literature search of the NCBI PubMed database (https://pubmed.ncbi.nlm.nih.gov/) using multiple keywords related to lung NE: “lung cancer” AND “lung neuroendocrine tumors” AND (“lung carcinoid” OR “small cell lung cancer” OR “lung large cell neuroendocrine tumors”) AND (“gene expression profiles” OR “RNA-seq data”). Datasets needed to fulfill the following criteria: 1) containing at least one NE subtype, or only containing non-NE subtypes but providing follow-up information; and 2) providing raw data or processed gene expression profiles with clear preprocessing and normalized methods. All datasets used in this study are displayed in Figure 1A, and the details are shown in Supplementary Table S1 (Supplementary Material).

The training dataset (GSE30219), including pathologically determined samples of 21 SCLC, 56 LCNEC, 24 CARCI, 85 ADC, and 61 SCC, was used to investigate the molecular landscape across lung cancer subtypes; data from 198 patients who had undergone only curative surgical resection were used for survival analysis. The dataset was further used as a training set to develop a panel of qualitative transcriptional signatures.

The qualitative signatures were tested step by step in 18 datasets that had fresh-frozen lung specimens, one dataset that had FFPE specimens, two datasets that had small biopsy specimens, two datasets that had mixed tumors with varied proportions of tumor cells, and one single-cell dataset, and these included 122 SCLC, 25 LCNEC, 137 CARCI, 6 NE, 2,155 ADC, 1,003 SCC, 4 adenosquamous carcinoma, and 12 other non-NE samples in total. LCC samples in these datasets, diagnosed according to the WHO 2004 criteria, were removed from this study since they might have included LCNEC samples. For the single-cell RNA-sequencing data (GSE131907), 32,764 tumor cells were derived from 58 samples of 44 ADC patients, including the primary tissues of 22 early-stage lung cancers (tLung) and advanced-stage lung cancers (tL/B), 17 brain metastases (mBrain) and metastatic lymph nodes (mLN) samples, and 5 pleural effusion samples.

In addition, among the 18 datasets with fresh-frozen samples, nine datasets (GSE42127, GSE50081, GSE37745, GSE26939, GSE31210, GSE31546, GSE14814, GSE17710, and GSE68465) with survival information were integrated for survival analysis, and these included 1,071 stage I–III ADC and SCC (non-NE) patients who had undergone only curative surgical resection. Supplementary Table S2 (Supplementary Material) shows the clinical information of these nine datasets.

For the microarray datasets generated by Affymetrix platforms, a robust multiarray average algorithm (Irizarry et al., 2003) was used for preprocessing the raw data. For the microarray datasets generated by Agilent and Illumina platforms, the originally processed data (series matrix files) were used with clear preprocessing and normalized methods. Probe IDs were mapped to gene IDs according to the corresponding platform files. For the RNA-sequencing datasets generated by Illumina Hiseq platforms, the originally processed data (series matrix files) were used. Ensembl gene IDs or gene symbols were mapped to the Entrez gene IDs.

### 2.2 Tissue samples, RNA extraction, and sequencing

Frozen biopsy specimens were obtained from 10 SCLC patients who underwent bronchoscopic intervention at the Harbin Medical University Cancer Hospital. Among them, nine patients were directly diagnosed by pathologists based on HE staining results, while one patient with a poorly differentiated tumor was further performed IHC detection for NE markers and finally diagnosed as SCLC by pathologists, which showed positivity for CD56, CgA, Syp, TTF-1, and CK7 and negativity for CK5/6 and P63. The samples were obtained under the ethical approval of the Institutional Review Boards of the Harbin Medical University Cancer Hospital, and written informed consent forms were obtained from all participants.

Total RNA was extracted according to the manufacturer’s protocol. The RNA quality was checked using Nanodrop (Thermo Company, United States). The purity and concentration of total RNA were determined using a Nanodrop spectrophotometer (Thermo Company, United States) according to the OD260/280 reading and a Qubit fluorescence quantifier (Invitrogen Company, United States), respectively. Paired-end sequencing with a read length of 100 bp was conducted using the Illumina Hiseq 2500/3000 platform (Illumina, San Diego, CA), and the final processed RNA-sequencing data were termed as SCLC data of Harbin Medical University (HMU-SCLC) (Figure 1A). Data and further clinical information are available from the corresponding author upon request.

### 2.3 Consensus clustering analyses

Consensus clustering was performed using the “ConsensusClusterPlus” package version 1.52.0 according to the Ward method for hierarchical clustering (Wilkerson and Hayes, 2010). The samples were clustered into *k* groups (*k* = 2–10) via Pearson’s correlation distance using the top 1,000 most variable genes across all samples in a cohort. The *k* value that corresponded to the first downward inflection in the cumulative distribution function was selected as the optimum number of clusters.

### 2.4 Hierarchical identification of qualitative signatures for lung cancer subtypes

A hierarchical rank-based method was developed to construct multiple qualitative signatures of lung cancer subtypes step by step.

#### 2.4.1 Identification of subtype candidate genes

To improve the accuracy of the training samples, dubious samples, whose consensus clustering results were discordant with their original pathological subtypes, were removed. Student’s *t* test was used to identify differentially expressed genes (DE genes) between the two clustering-adjusted subtype groups. The *p* values were adjusted using the Benjamini–Hochberg method for multiple testing to control the false discovery rate (FDR) (Benjamini and Hochberg, 1995). Genes with FDR of < 5% were defined as DE genes. As genes with larger differences between the two subtype groups provide more effective classification information, the top 1,000 DE genes with the largest fold changes (FCs) were selected as “candidate genes.”

#### 2.4.2 Identification of reversed gene pairs between the two subtypes

For a pair of genes, G*a* and G*b*, derived from the candidate genes, Fisher’s exact test was used to assess whether the frequency of a specific REO pattern (E*a* > E*b* or E*a* ≤ E*b*) in one clustering-adjusted subtype sample was significantly different from that in another clustering-adjusted subtype sample. Here, E*a* and E*b* are the gene expression values of G*a* and G*b*, respectively. Gene pairs with FDR of < 5% were defined as significantly reversed gene pairs between the two subtypes.

#### 2.4.3 Construction of gene pair signature for the two subtypes

A gene pair signature was constructed from all significantly reversed gene pairs as follows: First, for each significantly reversed gene pair, its classification consistency with pathologically diagnostic subtypes was calculated. Here, the classification consistency was termed as the “apparent” accuracy, since the pathological assessments were not 100% reliable and there may be misclassified cases according to clinical pathological methods.

Apparent   accuracy  =  SN × 100 (1)

where *S* is the number of samples whose classification subtypes predicted by the gene pair (G*a* and G*b*) were consistent with their original pathological subtypes, and *N* is the total number of corresponding subtype samples used in the dataset.

Second, all the significantly reversed gene pairs were chosen as an initial set, and all the genes contained in the initial set were used as seed. Then, a de-redundant method was utilized to obtain an optimal gene pair set based on the filter rule as follows: For any gene in the seed, if there were multiple gene pairs containing the gene, the one with the highest apparent accuracy was retained. If multiple gene pairs achieved the same maximum apparent accuracy, the gene pair with the largest absolute rank difference (Eq. 2) between the two subtypes was retained. By traversing all genes in the seed and removing the redundant gene pairs, we finally obtained an optimal gene pair set. This improves their robustness to batch effects and quality uncertainties of the clinical samples.

$$\bar{R_{\mathrm{ab}}}\;=\;\sqrt{\bar{R_{\mathrm{ab}\left(g1\right)}}\times \bar{R_{\mathrm{ab}\left(g2\right)}}}$$ (2)

where $\bar{R_{\mathrm{ab}\left(g1\right)}}$ and $\bar{R_{\mathrm{ab}\left(g2\right)}}$ are the geometric means of the absolute rank differences of the gene pair (G*a* and G*b*) in all samples between the two subtype groups (*g*1 and *g*2), respectively.

At last, the classification score for each sample was calculated as the sum of the classification votes of all the gene pairs in the set. The majority voting rule of the reversed gene pairs within a sample was adopted for classification, where if more than half of the gene pairs within the sample voted for one subtype, the sample was classified into that subtype.

In the training dataset (GSE30219), the above method was utilized to develop the NEsubtype-panel composed of three transcriptional signatures to distinguish the NE from non-NE tumors, CARCI from non-CARCI tumors, and SCLC from LCNEC tumors. To improve the robustness of the signatures to RNA degradation or low RNA input, which usually occur in clinically challenging samples, such as FFPE and biopsy samples, the gene pairs that have the gene with undetected expression value were removed and the majority voting rule of the remaining gene pairs in the signature was adopted for classification.

### 2.5 Functional enrichment, differential, and survival analyses

“ClusterProfiler” R package (Yu et al., 2012) was performed to conduct the functional enrichment analyses based on the current Gene Ontology databases, where a hypergeometric test was used.

Analysis of variance (ANOVA) was used to test the differences across multiple groups. RankProd (RP) algorithm of the “RankProd” R package version 3.14.0 (Hong et al., 2006), a nonparametric test, was conducted to estimate whether the subtype-specific marker genes were differentially expressed between the signature-confirmed and reclassified samples. The subtype-specific marker genes contain three NE marker genes (*CD56*, *SYP*, and *CHGA*) (Karlsson et al., 2017), two SCC marker genes (*KRT5* and *TP63*), and one ADC marker gene (*NAPSA*) (Kim et al., 2013). Here, a commonly used ADC marker gene (*TTF-1*) was excluded, since it is also highly expressed in partial SCLC samples (Rekhtman, 2022). Wilcoxon rank-sum test was used to test the difference in proliferation scores between the signature-confirmed and reclassified samples.

Overall survival (OS) is defined as the time from the date of initial surgical resection to the date of death or last contact (censored). To avoid deviations in the patient follow-up time among the different datasets, patient OS was truncated at 60 months. Survival curves were estimated using the Kaplan–Meier method and were statistically compared using the log-rank test (Bland and Altman, 2004). A multivariate Cox proportional-hazards regression model was used to assess whether the reclassified groups were independently associated with the patient survival after adjusting for data centers and clinical parameters, such as tumor stage, age, and sex. Hazard ratios (HRs) and 95% confidence intervals (CIs) were generated using univariate and multivariate Cox proportional-hazards models.

All statistical analyses were conducted using R 3.6.2 software (http://www.r-project.org/). Significance was defined as *p* < 0.05 or FDR < 0.05 for multiple testing.

## 3 Results

### 3.1 Transcriptional characteristics of lung cancer subtypes

The clinical and transcriptional characteristics of 247 lung cancer samples in the GSE30219 dataset were investigated and are displayed in Figure 1B. The different lung cancer subtypes represent diverse demographic and clinical characteristics and mRNA expression levels of subtype-specific marker genes. The proliferation scores, stemness scores, and hypoxia scores were estimated based on the mRNA expression profiles (Supplementary Material). The SCLC subtype showed the highest proliferation and stemness scores, followed by the LCNEC subtype (ANOVA, *p* < 0.0001, Figure 1B, Supplementary Figures S1A,B), suggesting a high grade of malignancy and poor differentiation. The SCC subtype had the highest hypoxia score, followed by the LCNEC and SCLC subtypes (ANOVA, *p* < 0.0001, Figure 1B, Supplementary Figure S1C). By contrast, CARCI exhibited the lowest proliferation, stemness, and hypoxia scores. Then, the immune landscape across lung cancer subtypes was depicted, including the immune scores calculated by ESTIMATE (Yoshihara et al., 2013), abundances of 28 subpopulations of infiltrating immune cells quantified by single-sample gene set enrichment analysis (Subramanian et al., 2005) (Supplementary Material), and mRNA expression levels of three immune checkpoint genes (*PD-1*, *PD-L1*, and *CTLA4*). The CARCI subtype was characterized by low levels of immune score, cell infiltration, and immune checkpoint gene expression, while partial LCNEC and SCLC samples showed high levels of the three immune indexes (ANOVA, *p* < 0.0001, Figure 1B, Supplementary Figure S1D), suggesting that these patients might benefit from immunotherapy. The survival analysis showed that SCLC and LCNEC patients had the worse prognoses, while CARCI patients had a favorable survival, when compared with ADC and SCC patients (log-rank *p* < 0.0001, Figure 1C). These results highlighted the discrepancies in tumor molecular biology across lung cancer subtypes.

**FIGURE 2:**
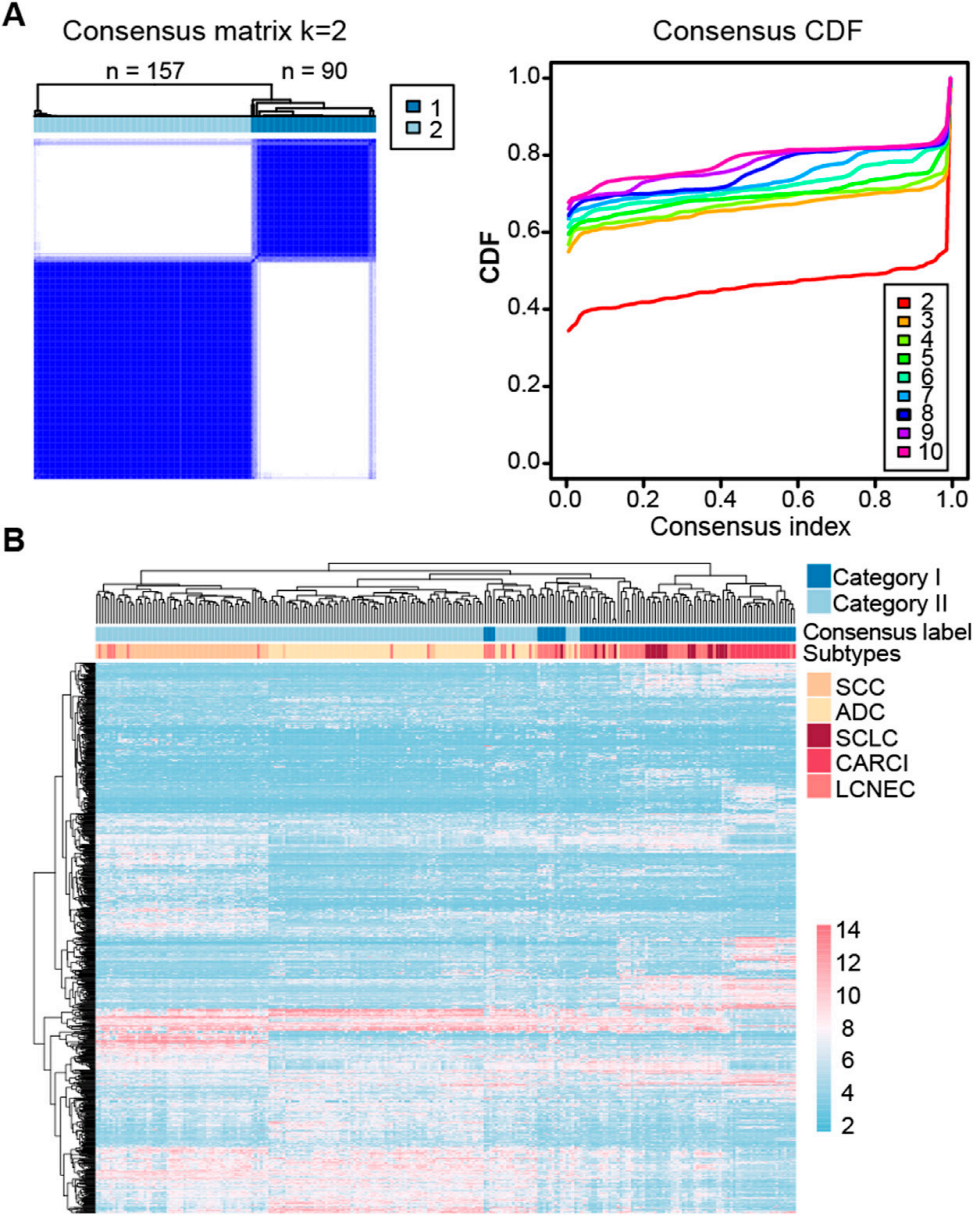
Clustering heatmap of lung cancer subtypes in the GSE30219 dataset. **(A)** consensus clustering of all the lung cancer samples based on the top 1,000 most variable genes in the dataset. The left panel represents the matrix heatmap when *k* = 2, and the right panel represents the consistent cumulative distribution function graph. **(B)** hierarchical clustering of all the samples based on the top 1,000 most variable genes.

At last, consensus clustering was performed for all samples in the GSE30219 dataset, and it was found that the samples were optimally classified into two subgroups (Figure 2A) with 157 and 90 samples, respectively, of which 87.13% of the NE samples were clustered into category I (named as NE-like), and 98.63% of the non-NE samples were clustered into category II (named as non-NE-like). The results indicated that NE (SCLC, LCNEC, and CARCI) and non-NE (ADC and SCC) samples had distinct transcriptional patterns. A similar result was observed after the hierarchical clustering (Figure 2B). It is worth noting that the hierarchical clustering result also showed that CARCI, SCLC, and LCNEC in the NE-like category had different gene expression patterns. These results suggested that the transcriptomic would be an effective tool to determine the histological subtype of lung cancer.

### 3.2 Identification of the NEsubtype-panel of transcriptional signatures for NE subtypes

**FIGURE 3:**
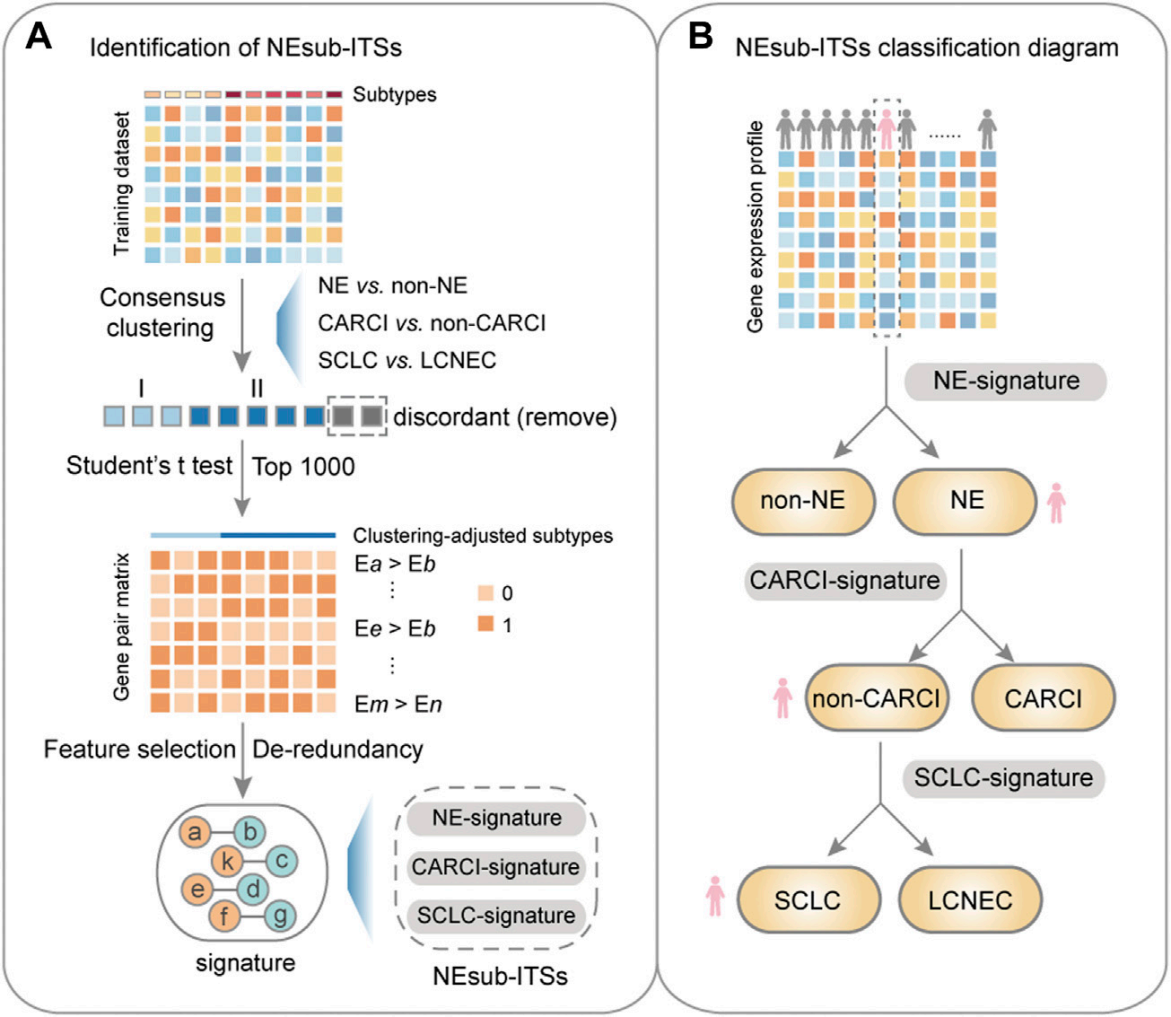
Flowchart of this study. **(A)** identification of the NEsubtype-panel. First, in the training dataset (GSE30219), a consensus clustering was performed based on mRNA expression to remove the discordant samples, and then, a panel of transcriptional signatures for determining NE subtype (NEsubtype-panel) in the clustering-adjusted samples was hierarchically developed, based on the “within-sample” relative expression orderings (REOs) of gene pairs to determine the lung NE subtypes. Second, the NEsubtype-panel was tested in multiple datasets with fresh-frozen, clinically challenging (FFPE and small biopsy specimens), and single-cell samples. At last, survival and differential expression analyses were conducted to indirectly support the reclassification indicated by these signatures. **(B)** the NEsubtype-panel classification diagram. For a given sample, the NEsubtype-panel was used to classify the histological subtype step by step based on the “within-sample” REOs of gene pairs, and to ultimately determine the patient subtype. NE, neuroendocrine; non-NE, non-neuroendocrine; and FFPE, formalin-fixed paraffin-embedded.

Figure 3A describes the flowchart for developing and validating the NEsubtype-panel for the diagnosis of lung NE subtypes. First, the abovementioned consensus clustering results of 15 samples (13 NE and 2 non-NE samples) in the training set were discordant with their original pathological subtypes (Figure 2B) and thus were deleted from the training set. From the remaining 232 samples, 13,216 DE genes between the clustering-adjusted 88 NE and 144 non-NE groups were extracted (Student’s *t* test, FDR < 0.05), which was more than the 12,917 DE genes extracted between the original pathologically determined subtypes (Student’s *t* test, FDR < 0.05). Furthermore, 92.44% of the 12,623 overlapped DE genes had a higher FC value than that in the original pathological subtypes, indicating the rationality of removing the dubious samples. From the 13,216 DE genes between the two clustering-adjusted subtype samples, the top 1,000 DE genes with a large FC difference were selected to construct gene pairs. Next, 373,502 NE-specific gene pairs were extracted, whose specific REO patterns (E*a* > E*b*) occurred more frequently in the clustering-adjusted NE samples than those in the clustering-adjusted non-NE samples (Fisher’s exact test, FDR < 0.05). For each NE-specific gene pair, if its REO in a sample was E*a* > E*b*, it voted the sample as NE, and *vice versa*. At last, the de-redundant method (see Materials and methods) was utilized to generate an optimal gene pair set consisting of 22 gene pairs (Table 1), which were selected as the NE-signature for distinguishing NE from non-NE tumors. According to the major classification rule, the apparent accuracy of the NE samples (named as apparent sensitivity) was 95.45%, and the apparent accuracy of the non-NE samples (named as apparent specificity) was 100%.

**TABLE 1:** Gene pair composition of the NEsubtype-panel

No.	NE-signature	CARCI-signature	SCLC-signature
	Gene *a* > Gene *b*	Gene *a* > Gene *b*	Gene *a* > Gene *b*
1	*KIF5C* (3800) > *CXCL2* (2920)	*NAP1L3* (4675) > *UBE2C* (11065)	*SEZ6L* (23544) > *ANG* (283)
2	*TMEM145* (284339) > *P2RY2* (5029)	*XKR4* (114786) > *NDC80* (10403)	*ATCAY* (85300) > *LOC100505490* (100505490)
3	*INSM1* (3642) > *TPSAB1* (7177)	*GAL3ST1* (9514) > *AURKA* (6790)	*PLCXD2* (257068) > *FAH* (2184)
4	*CAMK2N2* (94032) > *KCNK6* (9424)	*ABAT* (18) > *CDCA5* (113130)	*ZNF711* (7552) > *SRXN1* (140809)
5	*LRRC49* (54839) > *EPHA2* (1969)	*CDO1* (1036) > *RAD51AP1* (10635)	*DBH-AS1* (138948) > *TRPM4* (54795)
6	*CELSR3* (1951) > *SGMS2* (166929)	*CTNNA2* (1496) > *NUF2* (83540)	*KCNC1* (3746) > *C4orf19* (55286)
7	*RAB39B* (116442) > *COL17A1* (1308)	*LOC100286909* (100286909) > *GPNMB* (10457)	*LOC284219* (284219) > *SLC12A8* (84561)
8	*ACYP1* (97) > *YAP1* (10413)	*ZNF540* (163255) > *AUNIP* (79000)	*CENPK* (64105) > *SLC50A1* (55974)
9	*UBE2QL1* (134111) > *ITGB6* (3694)	*MTMR11* (10903) > *UHRF1* (29128)	*DPYSL5* (56896) > *SERPINA3* (12)
10	*PTPRN* (5798) > *AREG* (374)	*LOC257396* (257396) > *E2F7* (144455)	*NFIB* (4781) > *NOTCH2* (4853)
11	*GNAZ* (2781) > *PRODH* (5625)	*USP27X-AS1* (158572) > *MCM6* (4175)	*BRSK2* (9024) > *ABCC4* (10257)
12	*MIR7-3HG* (284424) > *SCEL* (8796)	*ITIH1* (3697) > *BUB1* (699)	*TMOD2* (29767) > *S100P* (6286)
13	*STMN3* (50861) > *C1orf116* (79098)	*SLC35F3* (148641) > *CDC6* (990)	*ST6GAL2* (84620) > *AJUBA* (84962)
14	*SH3GL2* (6456) > *SFTA2* (389376)	*TCEAL2* (140597) > *RFC4* (5984)	*ELAVL3* (1995) > *ADA* (100)
15	*CENPV* (201161) > *CARD6* (84674)	*NAP1L2* (4674) > *CAPG* (822)	*MRAP2* (112609) > *ACP6* (51205)
16	*ST18* (9705) > SH3RF2 (153769)	*ZNF658* (26149) > *SYK* (6850)	*FBXO43* (286151) > *GSTM4* (2948)
17	*RAB3B* (5865) > *KRT16* (3868)	*CCDC184* (387856) > *DEPDC1B* (55789)	*C5orf49* (134121) > *CTAG2* (30848)
18	*NRCAM* (4897) > *TMPRSS4* (56649)	*RGS11* (8786) > *PARPBP* (55010)	*DAND5* (199699) > *PDP2* (57546)
19	*BEX2* (84707) > *TNFSF10* (8743)	*LOC100130360* (100130360) > *SKP2* (6502)	*LRFN5* (145581) > *GTSF1* (121355)
20	*SCN3A* (6328) > *SLC6A14* (11254)	*MNX1-AS1* (645249) > *CENPF* (1063)	*LOC284244* (284244) > *KCNE4* (23704)
21	*SOWAHA* (134548) > *ACE2* (59272)	*SLC22A17* (51310) > *EZH2* (2146)	*ASPM* (259266) > *SPATC1L* (84221)
22	*PEG10* (23089) > *CEACAM6* (4680)	*SYT5* (6861) > *E2F8* (79733)	*CACNA1A* (773) > *C15orf48* (84419)
23		*NRXN1* (9378) > *KIF14* (9928)	*LRRC75A* (388341) > *TIMP3* (7078)
24		*SPRYD7* (57213) > *TTK* (7272)	*KIRREL3* (84623) > *TRIM6* (117854)
25		*PPP1R1A* (5502) > *KIT* (3815)	*KIF28P* (100130097) > *ME1* (4199)
26		*MYT1L* (23040) > *CNTNAP2* (26047)	*LMO2* (4005) > *PCOLCE* (5118)
27		*C5* (727) > *SLC7A5* (8140)	*ADAM22* (53616) > *MAGEA1* (4100)
28		*MIA2* (117153) > *LCAL1* (80078)	*AMER2* (219287) > *AZGP1* (563)
29		*RGS7BP* (401190) > *SCGB2A1* (4246)	*ENHO* (375704) > *TMEM45A* (55076)
30		*RFX6* (222546) > *PDK4* (5166)	*STXBP5L* (9515) > *PRR15* (222171)
31			*DCC* (1630) > *VTCN1* (79679)
32			*SHD* (56961) > *CHSY3* (337876)
33			*ATP6V1FNB* (100130705) > *OLR1* (4973)
34			*PCDH8* (5100) > *MX2* (4600)
35			*FGF14* (2259) > *MUC13* (56667)
36			*SETBP1* (26040) > *IER3* (8870)
37			*SBK1* (388228) > *DSG2* (1829)
38			*EEF1A2* (1917) > *MXRA5* (25878)
39			*CNPY1* (285888) > *RFX4* (5992)
40			*ISL1* (3670) > *CHN2* (1124)

Gene Symbol and Entrez gene IDs (within brackets) are provided in Table 1. For each gene pair (Gene *a* and Gene *b*) in the NEsubtype-panel, if the expression of Gene *a* is greater than Gene *b* in a sample, then it was supported to classify the sample as NE, CARCI, or SCLC, respectively. NE, neuroendocrine; CARCI, carcinoids; and SCLC, small-cell lung cancer.

Second, consensus clustering for the 88 NE samples in the training cohort was performed (Supplementary Figure S2A), and it was found that CARCI samples had considerably different gene expression patterns from those of the SCLC and LCNEC samples (non-CARCI). By comparing the clustering results and original pathological subtypes, five discordant samples were deleted, and 11,682 DE genes between the clustering-adjusted CARCI and non-CARCI groups were extracted. Likewise, 305,986 CARCI-specific gene pairs were extracted, whose REO patterns in the CARCI samples were significantly different from those in non-CARCI samples (Fisher’s exact test, FDR < 0.05), and the CARCI-signature consisting of 30 non-redundant gene pairs was developed (Table 1). According to the major classification rule, the apparent accuracies for clustering-adjusted CARCI and non-CARCI samples were both 100%.

At last, for the 19 SCLC and 42 LCNEC samples, 15 discordant samples were deleted based on their consensus clustering (Supplementary Figure S2B), and the SCLC-signature consisting of 40 gene pairs was developed (Table 1). The apparent sensitivity and specificity for 13 clustering-adjusted SCLC and 33 LCNEC samples were both 100%.

Overall, the NEsubtype-panel is composed of the NE-signature, CARCI-signature, and SCLC-signature for determining NE subtypes step by step (Figure 3B). The R code for classification of the NEsubtype-panel is detailed in Supplementary R function (Supplementary Material).

**FIGURE 4:**
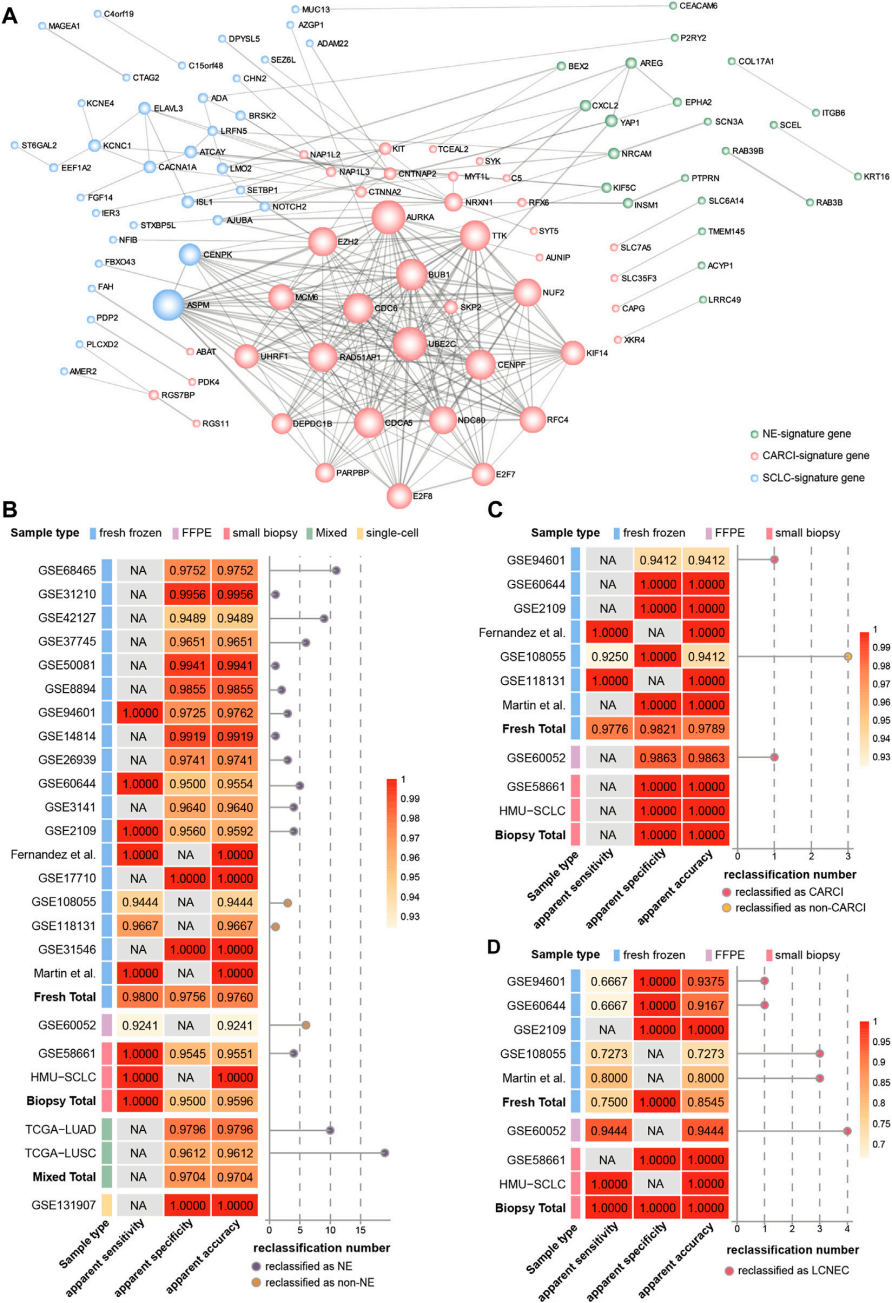
Hierarchical validation of the NEsubtype-panel. **(A)** protein–protein interaction network of genes in the NEsubtype-panel constructed using Cytoscape. The NE-signature, CARCI-signature, and SCLC-signature genes are marked in light green, pink, and blue, respectively. Line thickness indicates the strength of data support (interaction score by STRING). The apparent sensitivity, apparent specificity, and apparent accuracy of the **(B)** NE-signature, **(C)** CARCI-signature, and **(D)** SCLC-signature in multiple datasets. The left panel of each signature represents the classification accuracy of different sample types, and the right panel displays the number of reclassified samples. NE, neuroendocrine; CARCI, carcinoids; and SCLC, small-cell lung cancer.

Furthermore, based on The Search Tool for the Retrieval of Interacting Genes database (STRING) database, genes in the three signatures were mapped into the protein–protein interaction (PPI) network (Figure 4A). Then, the Cytoscape plug-in Molecular Complex Detection was applied to detect notable modules, and then, the function of these key genes was analyzed. For instance, for the CARCI-signature module, the gene set functions mainly involved cell division and mitotic spindle organization, corresponding to 10 genes downregulated in CARCI samples, which were supported by the knowledge that the mitotic index of CARCI is lower than that of SCLC and LCNEC (Righi et al., 2017). Besides, *YAP1* is overexpressed in NSCLC and the loss of *YAP1* has potential as a clinical marker for predicting NE features (Ito et al., 2016), and *YAP1*, combined with *ASCL1*, *NEUROD1*, and *POU2F3*, can be used to define SCLC subtypes (Baine et al., 2020). It is worth noting that the REO of two genes in a gene pair has intuitive biological implications in tumor subtype development. For instance, in gene pair *RAB3B-KRT16* in the NE-signature of the panel, *RAB3B* is a *Ras* oncogene superfamily member that controls the regulated exocytosis in neuronal/secretory cells, and its expression is significantly higher in NE (SCLC) samples than in non-NE (ADC, SCC, and LCC) samples (Zhang et al., 2016); however, keratin 16 (*KRT16*) is a type I cytokeratin, whose overexpression promotes tumorigenicity in ADC (Yuanhua et al., 2019). The relative order of *RAB3B* expression tended to be higher than that of *KRT16* in NE patients and was reversed in non-NE patients. In addition, hub genes with a higher degree in the network may be potential key therapeutic targets for NE subtypes. For example, the abnormal spindle-like, microcephaly associated (*ASPM*) with the highest degree in SCLC-signature was essential for normal mitotic spindle function-dependent cell division (Higgins et al., 2010; Zhang et al., 2015). Besides, Iwakawa et al. revealed that *ASPM* was frequently mutated in SCLC (Iwakawa et al., 2015). Our results showed that *ASPM* was significantly higher expressed in SCLC than in LCNEC (Student’s *t* test, *p* < 0.0001), indicating that *ASPM* might be a therapeutic target for SCLC (Zhang et al., 2015).

Therefore, PPI network construction and functional analyses of genes in the three transcriptional signatures provided biological evidences for their ability to determine the histological classification and clues for the treatment of lung cancer.

### 3.3 Hierarchical validation of the NEsubtype-panel

The NEsubtype-panel was tested on multiple independent lung cancer datasets. First, the NE-signature in the panel was tested on 18 fresh-frozen tissue datasets, including 200 NE and 2,048 non-NE samples (Figure 4B). In total, the apparent sensitivity of NE samples was 98.00%, the apparent specificity of non-NE samples was 97.56%, and the apparent accuracy was 97.60%. Likewise, in one dataset with FFPE specimens (GSE60052), 73 of 79 NE samples were confirmed by the signature, and the apparent sensitivity of the NE samples was 92.41%. In one dataset with small biopsy specimens (GSE58661) that had one NE and 88 non-NE samples, the apparent sensitivity for NE samples was 100%, the apparent specificity for non-NE samples was 95.45% (84/88), and the apparent accuracy was 95.51%. Likewise, we applied the NE-signature to mixed tumor samples with 10–100% tumor cells in TCGA-LUAD and TCGA-LUSC datasets. The overall apparent accuracies of the NE-signature for 490 ADC samples and 490 SCC samples were 97.96 and 96.12%, respectively. In the single-cell RNA-sequencing dataset (GSE131907) with 58 ADC samples, the apparent specificity for non-NE samples was 100% across all the 32,764 primary and metastatic tumor cells sampled from biopsy or pleural effusion.

Then, the classification accuracy of the CARCI-signature in the panel was verified in the 280 signature-confirmed NE samples across nine validation datasets (Figure 4C). The apparent sensitivity for CARCI samples reached 97.76% (131/134), and the apparent specificity for non-CARCI samples was 98.21% (55/56) in fresh-frozen specimens, 98.63% (72/73) in the one FFPE dataset (GSE60052), and 100% in one biopsy tissue dataset (GSE58661).

Next, the SCLC-signature in the panel was validated in the signature-confirmed non-CARCI samples (Figure 4D). The apparent sensitivity for SCLC samples was 75.00% (24/32), the apparent specificity for LCNEC was 100%, and the apparent accuracy was 85.45% in fresh-frozen specimens. In GSE60052, the dataset with FFPE specimens, 68 of 72 SCLC samples were confirmed by the signature, and the apparent accuracy was 94.44%. For two small biopsy specimen datasets, the apparent sensitivity for SCLC samples was 90.00% (9/10), all LCNEC samples were confirmed by the signature (1/1), and the apparent accuracy was 90.91%.

At last, we collected 10 SCLC biopsy samples from the clinic (HMU-SCLC), and the NEsubtype-panel exhibited 100% accuracy for these samples, indicating its clinical feasibility.

In total, the NEsubtype-panel had a good performance in distinguishing NE tumors from non-NE tumors and determined the NE subtypes not only in fresh-frozen specimens but also in samples with RNA degradation (FFPE) and low RNA input (small biopsy and single-cell specimens).

### 3.4 Biological analyses for reclassification

**FIGURE 5:**
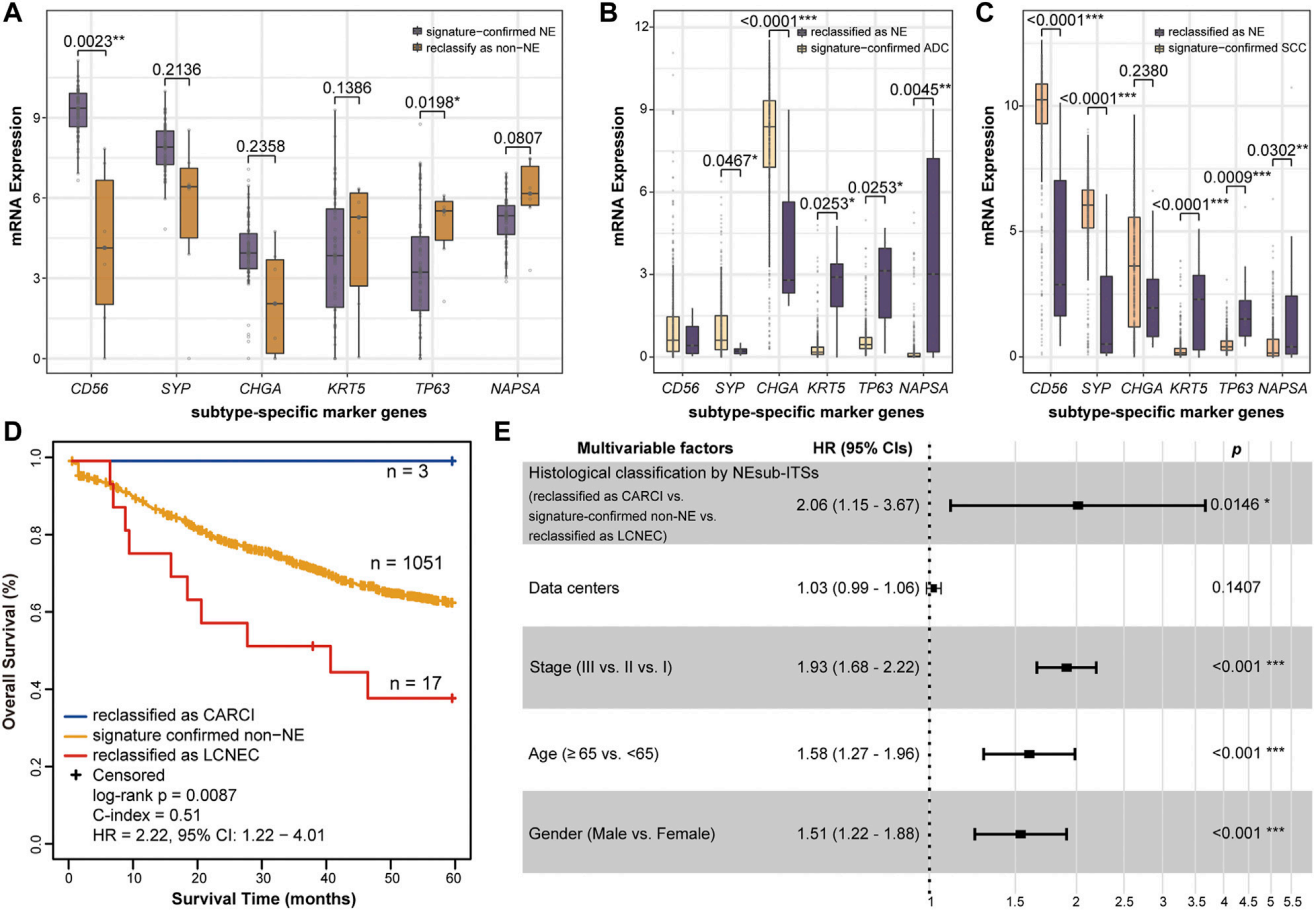
Biological analyses of the reclassification of the NEsubtype-panel. The boxplots of mRNA expression of the subtype-specific marker genes in **(A)** GSE60052, **(B)** TCGA-LUAD, and **(C)** TCGA-LUSC datasets with the most reclassified samples (6, 10, and 19 samples, respectively). The subtype-specific marker genes include three neuroendocrine marker genes (*CD56*, *SYP*, and *CHGA*), two SCC marker genes (*KRT5* and *TP63*), and one ADC marker gene (*NAPSA*). The RankProd algorithm was used to test the difference in the subtype-specific marker genes between the reclassified samples and the signature-confirmed samples. **(D)** Kaplan–Meier curves of overall survival for the non-NE samples that were reclassified as CARCI (blue), signature-confirmed non-NE (yellow), and non-NE samples reclassified as LCNEC (red). **(E)** multivariate Cox regression analysis for histological classification by signatures after adjusting for data center and clinical parameters in the integrated dataset. NE, neuroendocrine; CARCI, carcinoids; ADC, adenocarcinoma; SCC, squamous carcinoma; and LCNEC, large-cell neuroendocrine carcinoma.

As the subjective diagnoses of HE staining or immunostaining results by pathologists may lead to some misclassified cases (Guo et al., 2021), several biological analyses were conducted to indirectly support the reclassification indicated by the signatures. First, according to the above results, it was found using the NE-signature that the three datasets, namely, GSE60052 (NE samples), TCGA-LUAD (non-NE samples), and TCGA-LUSC (non-NE samples), had the most misclassified samples (6, 10, and 19, respectively). As a consequence, differential expression analyses were conducted for six subtype-specific marker genes. In the GSE60052 dataset, out of 73 signature-confirmed NE samples, six reclassified non-NE samples had significantly decreased expression of one NE marker gene (RP algorithm, *CD56*: *p* = 0.0023, Figure 5A) and significantly increased expression of one SCC marker gene (RP algorithm, *TP63*: *p* = 0.0198, Figure 5A). In the TCGA-LUAD dataset, the NEsubtype-panel reclassified 10 (2.04%) ADC samples as LCNEC, which had significantly increased expression of three NE marker genes and significantly decreased expression of the ADC marker gene, respectively, when compared with the signature-confirmed ADC samples (RP algorithm, *CD56*: *p* = 0.0253; *SYP*: *p* = 0.0253; *CHGA*: *p* = 0.0045; *NAPSA*: *p* < 0.0001, Figure 5B). Likewise, in the TCGA-LUSC dataset, compared with the signature-confirmed SCC samples, the 19 SCC samples reclassified as one CARCI and 18 LCNEC exhibited significantly increased expression of three NE marker genes (RP algorithm, *CD56*: *p* < 0.0001; *SYP*: *p* = 0.0009; *CHGA*: *p* = 0.0302, Figure 5C) and significantly decreased expression of two SCC marker genes (RP algorithm, *KRT5*: *p* < 0.0001; *TP63*: *p* = 0.0001, Figure 5C).

Next, the accuracy of reclassification by these signatures was further evaluated through survival analyses. Nine datasets were integrated and included 1,071 stage I–III ADC and SCC (non-NE) patients who were treated with only curative surgical resection and recorded survival information. From all the non-NE samples, 1,051 patients were confirmed by the NE-signature, and 20 patients were reclassified as NE, of which 3 and 17 cases were further reclassified as CARCI and LCNEC, respectively, by the CARCI- and SCLC-signatures. As expected, survival analysis showed that the three reclassified CARCI patients had significantly longer OS, while the 17 reclassified LCNEC patients showed significantly shorter OS than the other ADC and SCC patients (log-rank *p* = 0.0087, HR = 2.22, 95% CI = 1.22–4.01, Figure 5D) (Vesterinen et al., 2018; Jiang et al., 2021). Multivariate Cox analysis showed that the reclassified patients also had significantly different OS than the signature-confirmed non-NE patients (*p* = 0.0146, HR = 2.06, 95% CI = 1.15–3.67, Figure 5E), after adjusting for data centers and clinical parameters.

The above biological results provided evidence that these signatures might rectify some misclassifications that occur during routine pathological assessments.

## 4 Discussion

This study investigated the transcriptional characteristics of lung cancer subtypes and demonstrated that the different lung cancer subtypes represented diverse degrees of malignancies, immune cell infiltration, and transcriptional patterns, highlighting the discrepancies in tumor biology across lung cancer subtypes. Utilizing transcriptional data, a panel of signatures for the individualized pathological diagnosis of lung NE tumor was developed. To our knowledge, this is the first report of a panel of transcriptional signatures that can distinguish NE from non-NE tumors and determine NE subtypes accurately. Because of the limited number of NE samples and the often misdiagnosed samples during pathological diagnosis, the consensus clustering method was first applied to eliminate the dubious samples whose expression patterns were discordant with their pathological subtypes. The results showed that after removing these dubious samples, the number of DE genes between the two clustering-adjusted subtypes increased, and the degree of difference also improved. These results support the rationality of deleting these dubious samples to improve the training accuracy.

We have developed the NEsubtype-panel, which can be used for identifying NE subtypes based on the within-sample REOs of gene pairs for individualized applications. The NEsubtype-panel was effectively verified in 23 public datasets from multiple platforms, including Affymetrix, Agilent, and Illumina, and the overall consistencies of the three signatures with pathologically diagnostic subtypes were 97.31%, 98.11%, and 90.63%, respectively, which can thus be used to assist the pathologist in classifying lung NE tumors. The ability of the NEsubtype-panel to reliably distinguish lung NE subtypes was validated in multiple tissue types, even for clinical challenging tissues (FFPE and biopsy). These results suggested the advantage of the subtype panel in clinical applications. It is worth noting that the overall apparent sensitivity of the SCLC-signature for SCLC was 88.60%, which did not seem to be perfect. As our results showed that SCLC displayed higher proliferation ability, the reclassified LCNEC samples had significantly lower proliferation abilities than the signature-confirmed SCLC samples in two of the three datasets (GSE108055, Martin et al., and GSE60052) (Wilcoxon rank-sum test, GSE108055: *p* = 0.0480, GSE60052: *p* = 0.0066, Supplementary Figure S3). As a result, we additionally collected 10 SCLC frozen biopsy samples from the clinic and verified the accuracy of the NEsubtype-panel, indicating its clinical feasibility. A previous study has published a lung subtype panel, including 57 genes (57-gene), for distinguishing lung cancer subtypes (Faruki et al., 2016). In a word, gene centroid was calculated for each of three subtypes (ADC, SCC, and NE), respectively. Correlations between a test sample and each gene centroid were calculated (Spearman’s rank correlation), and then, the sample was assigned to a specific subtype (ADC, SCC, or NE) corresponding to the maximally correlated centroid. We compared with 57-gene in all the fresh-frozen and FFPE datasets in this study, and the results showed that the overall apparent accuracies were lower than that of the NE-signature in the panel in 15 frozen datasets and one FFPE dataset and equal to our signature in three frozen datasets (Supplementary Figure S4), indicating a superior performance of our developed the NEsubtype-panel. Moreover, another limitation of 57-gene is that it cannot be applied to small biopsy samples for subtype classification, while our panel can classify biopsy samples more accurately.

The overall classification accuracy of the NEsubtype-panel was high; however, the comparison of the classification performance between the NEsubtype-panel and NE immunomarkers (CgA, Syp, CD56, etc.) still deserved follow-up study. Although the accuracy of the NEsubtype-panel could reach more than 92%, there was still a certain percentage of discordant samples identified by pathological diagnosis and the NEsubtype-panel, which may lead to some misclassification because of subjective diagnosis of HE staining or immunostaining results by pathologists. The subtype-specific marker genes analysis provided transcriptional evidence to support the reclassifications obtained by our panel. Further, the reclassification of these signatures was supported using survival analyses by the knowledge that LCNEC patients have poorer prognoses and CARCI patients have better prognoses than those ADC and SCC patients. Such biological evidences support the classification accuracy of the NEsubtype-panel.

However, there are still some limitations of this study. One limitation is that the NEsubtype-panel could not distinguish between typical and atypical CARCIs in the CARCI samples because the samples of these two subtypes are associated with a low incidence of lung cancer, and thus, there are very few samples present currently to develop robust signatures. Another limitation is that most samples in the public datasets are diagnosed according to the WHO 2004 criteria, which might not be detected by IHC and needs further validation based on the samples diagnosed using the WHO 2015 criteria.

## 5 Conclusion

The novel transcriptional NEsubtype-panel, consisting of three gene pair signatures, was developed that could effectively distinguish lung NE tumors from non-NE tumors and determine the NE subtypes individually, even in clinically challenging samples (FFPE and biopsy samples). The combination of these signatures with our previously published signature (*KRT5* and *AGR2*) used for distinguishing SCC from non-SCC (ADC) samples could be used as an RNA-sequencing panel to complement the morphology-based classification of lung tumors. This would also help in preserving precious tissue samples that can then be used for conducting other molecular tests.

## Data Availability

The RNA-sequencing data (HMU-SCLC) presented in the study are deposited in The Genome Sequence Archive for Human repository (GSA-Human, http://bigd.big.ac.cn/gsa-human), accession number HRA000516. Data and further clinical information are available from the corresponding author upon request.

## Abbreviations

NE, neuroendocrine; non-NE, non-neuroendocrine; HE, hematoxylin-eosin; LCC, large-cell carcinoma; CHGA/CgA, chromogranin; SYP/Syp, synaptophysin; REOs, relative gene expression orderings; HMU-SCLC, SCLC data of Harbin Medical University; DE genes, differentially expressed genes; and RP, RankProd.
