# Supplementary material for: Hierarchical identification of a transcriptional panel for the histological diagnosis of lung neuroendocrine tumors
Source: Front Genet. 2022 Aug 29;13:944167. doi: 10.3389/fgene.2022.944167 (PMC9465419; doi:10.3389/fgene.2022.944167)
Supplement: Supplementary file 1 [file Table1.docx]

Supplementary Material

# Supplementary Methods

The proliferation scores, stemness scores, hypoxia scores, immune scores, and immune cell infiltrations of samples were calculated according to their algorithms described in their original literatures, which were briefly described in the Supplementary Methods.

**Proliferation score.** The proliferation score for a given sample was calculated by averaging the mRNA expression levels of 44 genes in the proliferation signature.([Whitfield et al., 2006](#_ENREF_23)) The high proliferation score represents high proliferative capacity.

**Stemness score.** The ssGSEA([Subramanian et al., 2005](#_ENREF_22)) on the 109 stemness-related gene signatures([Miranda et al., 2019](#_ENREF_19)) was performed to calculate the stemness score by using GSVA package in R. The high score represents high stemness of the tumours.

**Hypoxia score.** The hypoxia score for a given sample is defined as the median expression of the 26 hypoxia genes.([Eustace et al., 2013](#_ENREF_7)) The hypoxia score is then normalised between 0 and 1, where 0 represents the least hypoxic and 1 represents the most hypoxic.

**Immune score and immune cell infiltration.** ESTIMATE([Yoshihara et al., 2013](#_ENREF_27)) was introduced to estimate the immune score for a given sample by performing ssGSEA([Subramanian et al., 2005](#_ENREF_22)) based on its mRNA expression profiles using “estimate” (R package). The ssGSEA([Subramanian et al., 2005](#_ENREF_22)) was also utilized to quantify the relative infiltration abundances of 28 immune cell types in the tumor microenvironment by using “GSVA” (R package). The relative infiltration abundance of each immune cell type was represented by an enrichment score in ssGSEA analysis.

# Supplementary Tables and Figures

## Supplementary Tables

**Table S1. The data sets of lung cancer used in this study**

| Data source | Platform | Tissue type | ADC | SCC | LCNEC | SCLC | CARCI | other |
| --- | --- | --- | --- | --- | --- | --- | --- | --- |
| GSE30219([Rousseaux et al., 2013](#_ENREF_21)) | Affymetrix Plus 2.0 | frozen | 85 | 61 | 56 | 21 | 24 |  |
| GSE68465([Director's Challenge Consortium for the Molecular Classification of Lung et al., 2008](#_ENREF_6)) | Affymetrix U133A | frozen | 443 | 0 | 0 | 0 | 0 |  |
| GSE31210([Yamauchi et al., 2012](#_ENREF_26)) | Affymetrix Plus 2.0 | frozen | 226 | 0 | 0 | 0 | 0 |  |
| GSE42127([Hight et al., 2020](#_ENREF_10)) | Illumina WG-6 v3.0 | frozen | 133 | 43 | 0 | 0 | 0 |  |
| GSE37745([Botling et al., 2013](#_ENREF_4); [Lohr et al., 2015](#_ENREF_18); [Jabs et al., 2017](#_ENREF_11)) | Affymetrix Plus 2.0 | frozen | 106 | 66 | 0 | 0 | 0 |  |
| GSE50081([Der et al., 2014](#_ENREF_5)) | Affymetrix Plus 2.0 | frozen | 127 | 43 | 0 | 0 | 0 |  |
| GSE8894([Lee et al., 2008](#_ENREF_17)) | Affymetrix Plus 2.0 | frozen | 63 | 75 | 0 | 0 | 0 |  |
| GSE94601([Karlsson et al., 2017](#_ENREF_13)) | Illumina HT-12 V4.0 | frozen | 83 | 26 | 14 | 3 | 0 |  |
| GSE14814([Zhu et al., 2010](#_ENREF_28)) | Affymetrix U133A | frozen | 71 | 52 | 0 | 0 | 0 |  |
| GSE26939([Wilkerson et al., 2012](#_ENREF_25)) | Agilent-UNC-custom-4X44K | frozen | 116 | 0 | 0 | 0 | 0 |  |
| GSE60644([Karlsson et al., 2014](#_ENREF_14)) | Illumina HT-12 V4.0 | frozen | 77 | 22 | 9 | 3 | 0 | 1ADSC |
| GSE3141([Bild et al., 2006](#_ENREF_3)) | Affymetrix Plus 2.0 | frozen | 58 | 53 | 0 | 0 | 0 |  |
| GSE2109 | Affymetrix Plus 2.0 | frozen | 45 | 41 | 1 | 0 | 0 | 2NSCLC;  3ADSC;  6NE |
| Fernandez *et al.*([Fernandez-Cuesta et al., 2014](#_ENREF_8)) | Illumina HiSeq 2000 | frozen | 0 | 0 | 0 | 0 | 65 |  |
| GSE17710([Wilkerson et al., 2010](#_ENREF_24)) | Agilent-UNC-custom-4X44K | frozen | 0 | 56 | 0 | 0 | 0 |  |
| GSE108055([Asiedu et al., 2018](#_ENREF_2)) | Illumina HumanWG-6 v2.0 expression beadchip | frozen | 0 | 0 | 0 | 12 | 42 |  |
| GSE118131([Laddha et al., 2019](#_ENREF_16)) | Illumina HiSeq 2000 | frozen | 0 | 0 | 0 | 0 | 30 |  |
| GSE31546 | Affymetrix Plus 2.0 | frozen | 17 | 0 | 0 | 0 | 0 |  |
| Martin *et al.*([Peifer et al., 2012](#_ENREF_20)) |  | frozen | 0 | 0 | 0 | 15 | 0 |  |
| GSE60052([Jiang et al., 2016](#_ENREF_12)) | Illumina HiSeq 2000 | FFPE | 0 | 0 | 0 | 79 | 0 |  |
| GSE58661([Aerts et al., 2014](#_ENREF_1); [Grossmann et al., 2017](#_ENREF_9)) | Affymetrix 2.0 | Biopsy | 42 | 36 | 1 | 0 | 0 | 10NSCLC |
| HMU-SCLC | Illumina HiSeq 2500/3000 | Biopsy* | 0 | 0 | 0 | 10 | 0 |  |
| TCGA-LUAD | Illumina HiSeqV2 | Mixed | 490 | 0 | 0 | 0 | 0 |  |
| TCGA-LUSC | Illumina HiSeqV2 | Mixed | 0 | 490 | 0 | 0 | 0 |  |
| GSE131907([Kim et al., 2020](#_ENREF_15)) | Illumina HiSeq 2500 | Single-cell RNA sequencing | 58 | 0 | 0 | 0 | 0 |  |

*Note*: *RNA-sequencing data for biopsy specimens generated in this study. FFPE, formalin fixed paraffin-embedded; ADSC, adenosquamous carcinoma; NSCLC, non-small cell lung cancer.

**Table S2.** **Baseline clinical characteristics of patients treated with curative surgery resection only**

| Data Source | Year | Sample | Age | | Gender | | Stage | | |
| --- | --- | --- | --- | --- | --- | --- | --- | --- | --- |
|  |  |  | Age < 65 | Age ≥ 65 | Female | Male | I | II | III |
| GSE42127 | 2013 | 122 | 50 | 72 | 62 | 60 | 83 | 23 | 16 |
| GSE50081 | 2013 | 170 | 55 | 115 | 80 | 90 | 119 | 51 | 0 |
| GSE37745 | 2012 | 64 | 22 | 42 | 33 | 31 | 46 | 8 | 10 |
| GSE26939 | 2012 | 85 | 42 | 43 | 44 | 41 | 55 | 16 | 14 |
| GSE31210 | 2011 | 204 | 145 | 59 | 109 | 95 | 162 | 42 | 0 |
| GSE31546 | 2011 | 13 | 13 | 0 | 10 | 3 | 13 | 0 | 0 |
| GSE14814 | 2010 | 58 | 40 | 18 | 17 | 41 | 32 | 26 | 0 |
| GSE17710 | 2010 | 56 | 22 | 34 | 24 | 32 | 34 | 19 | 3 |
| GSE68465 | 2008 | 299 | 132 | 167 | 145 | 154 | 211 | 51 | 37 |

## Supplementary Figures

**
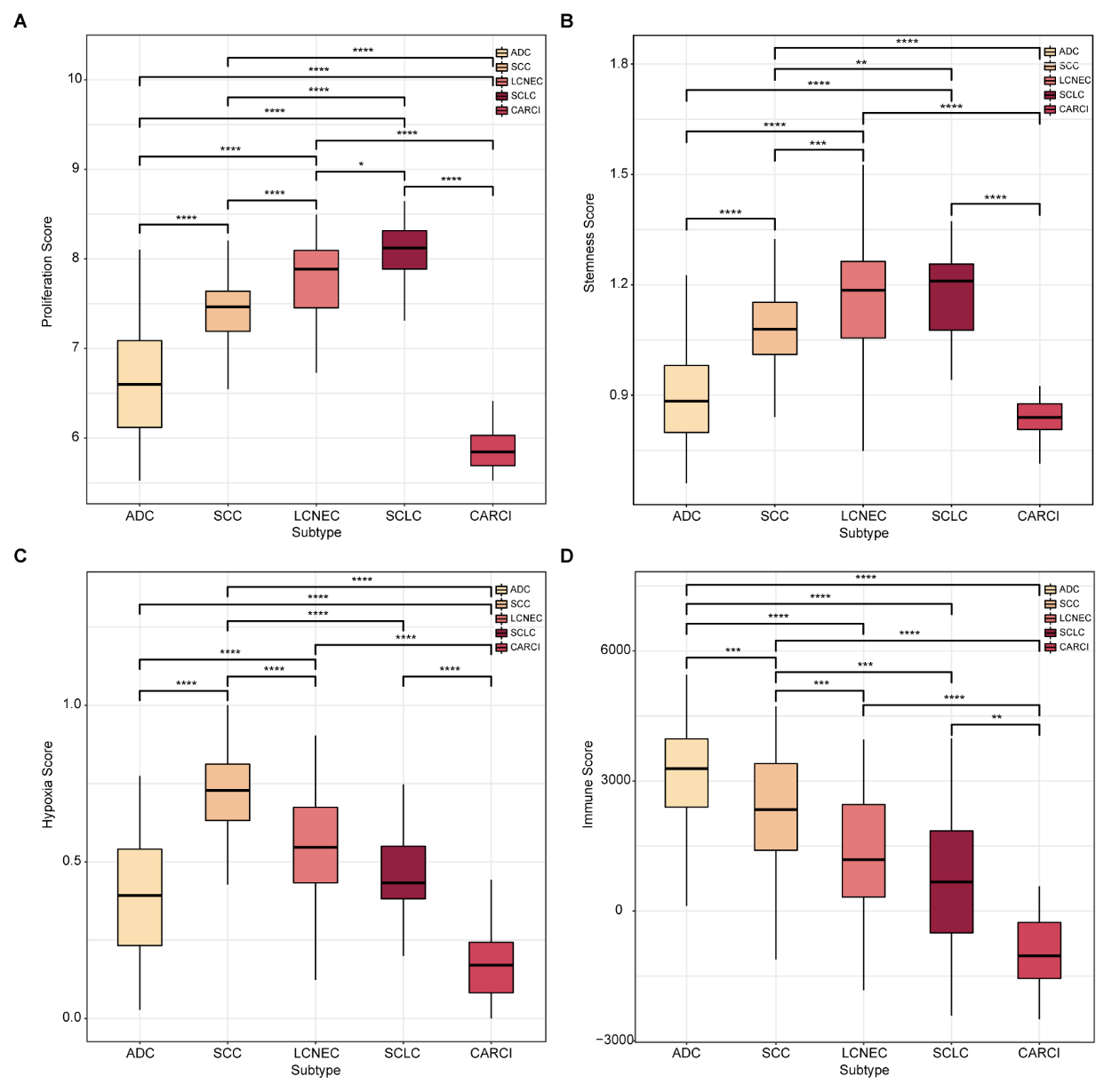
**

**Supplementary Figure S1. The boxplots of** (**A**) **proliferation scores,** (**B**) **stemness scores,** (**C**) **hypoxia scores and** (**D**) **immune scores across lung cancer subtypes.** Wilcoxon rank sum test was used to test the difference of the above four scores between two groups. ADC, adenocarcinoma; SCC, squamous carcinoma; LCNEC, large-cell neuroendocrine carcinomas; SCLC, small-cell lung cancer; CARCI, carcinoids.

**
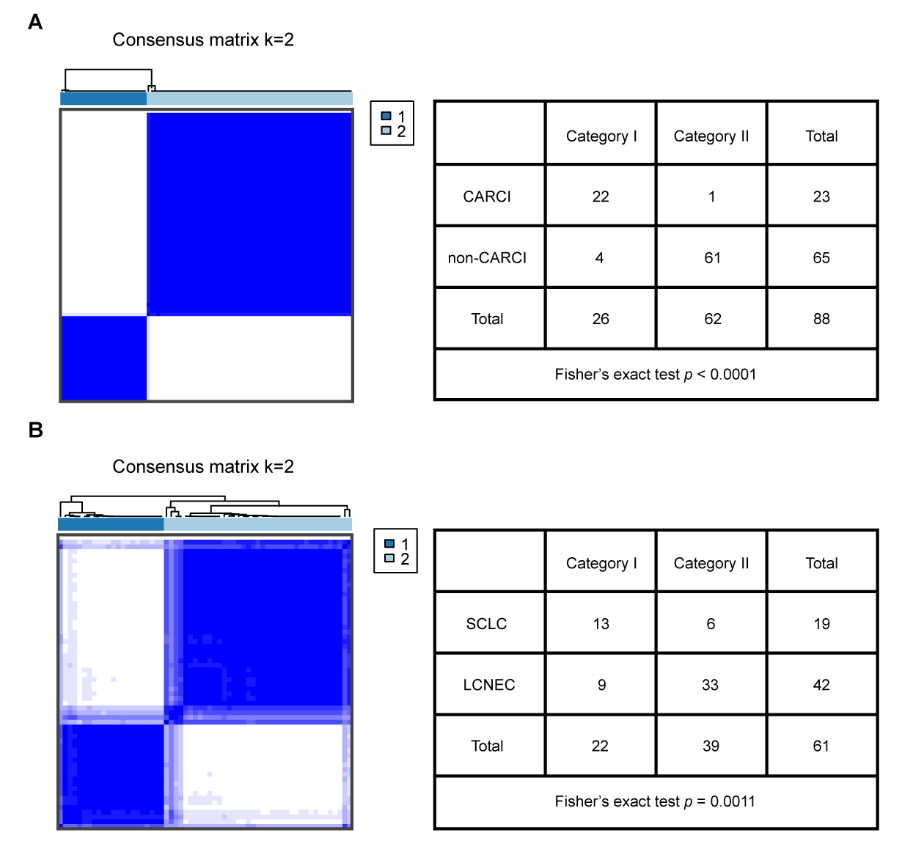
**

**Supplementary Figure S2. Clustering heatmap and sample distribution of lung cancer subtypes in the GSE30219 dataset**

(**A**) Consensus clustering of all the NE samples based on the top 1,000 most variable genes (left) and clustering sample distribution table (right) for each category. (**B**) Consensus and hierarchical clustering of all the SCLC and LCNEC samples based on the top 1,000 most variable genes. Fisher’s exact test was used to compare the overlap between the two categories of consensus clustering and two lung cancer subtypes. NE, neuroendocrine; SCLC, small-cell lung cancer; LCNEC, large cell neuroendocrine.

**
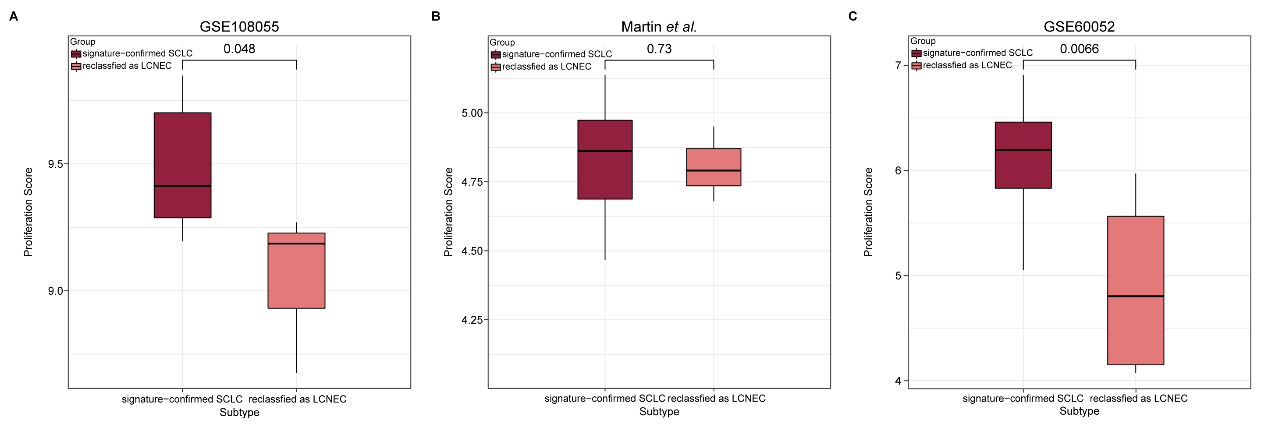
**

**Supplementary Figure S3. The boxplots of the proliferation scores between signature-confirmed SCLC samples and re-classified as LCNEC samples in** (**A**) GSE108055, (**B**) Martin *et al.*, (**C**) GSE60052 datasets. Wilcoxon rank sum test was used to test the difference of the proliferation scores between two groups. SCLC, small-cell lung cancer; LCNEC, large-cell neuroendocrine carcinomas.


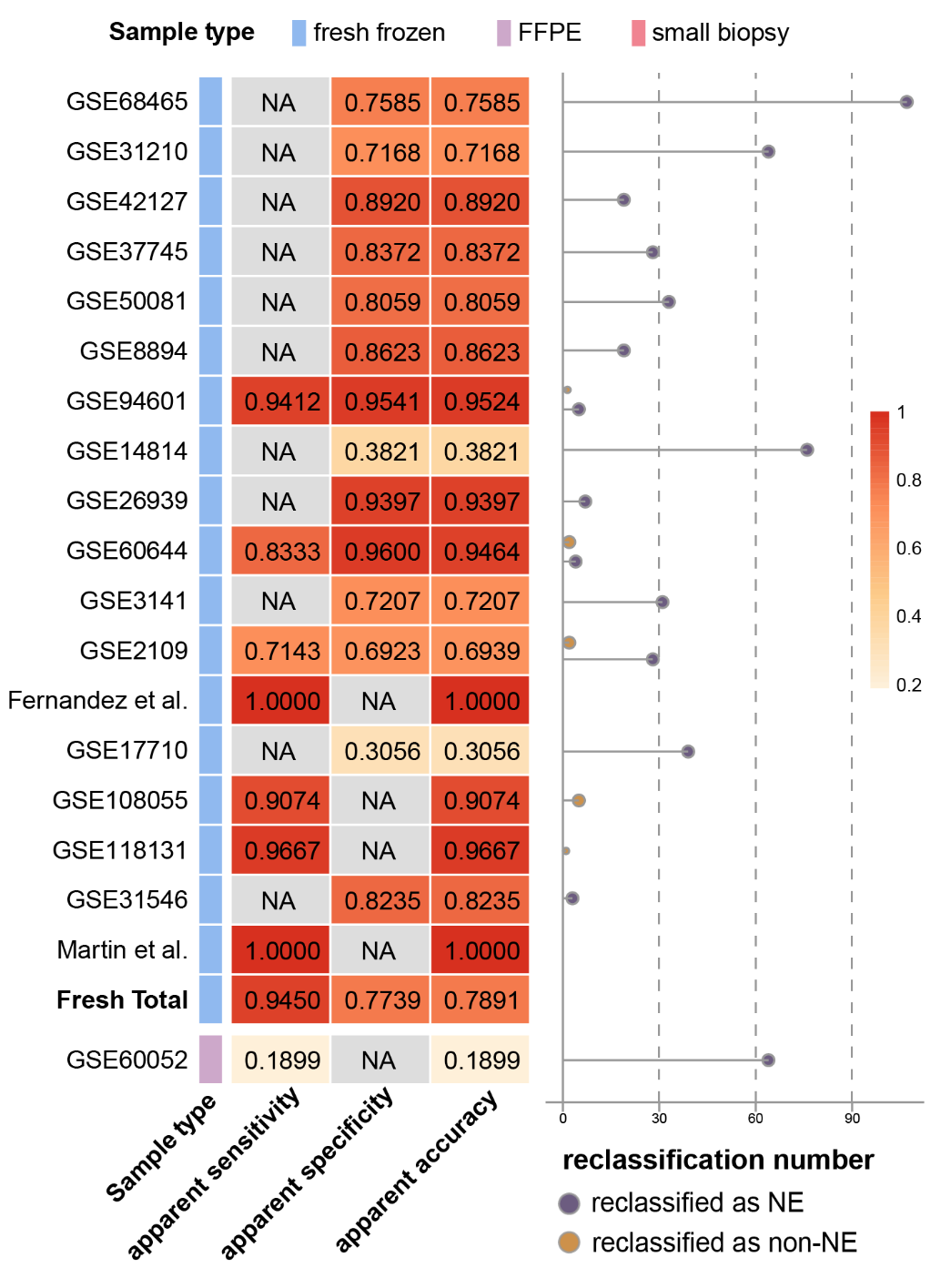


**Supplementary Figure S4. Validation of the 57-gene.** The apparent sensitivity, apparent specificity, and apparent accuracy of the 57-gene in multiple datasets. The left panel represents the classification accuracy of different sample types, and the right panel displays the number of re-classified samples. NE, neuroendocrine.

# Supplementary R function

# Description

# A panel of transcriptional signatures to determine lung neuroendocrine subtypes (NEsubtype-panel) for an individual

#

# Input

# exp: A matrix of expression profiles

# geneid: A vector of gene list

#

# Output

# The predicted lung neuroendocrine specific subtypes for lung patients.

#

# Examples

# subResults <- sub_class(exp,geneid,pair_ne,pair_carci,pair_sclc)

#####################################################################

R code:

pair_ne <- read.table("NE_signature.txt",sep='\t',header=T) #The file ' NE_signature.txt' included the signature genes could be found in Table 1.

pair_carci <- read.table("CARCI_signature.txt",sep='\t',header=T) #The file ' CARCI _signature.txt' included the signature genes could be found in Table 1.

pair_sclc <- read.table("SCLC_signature.txt",sep='\t',header=T) #The file ' SCLC _signature.txt' included the signature genes could be found in Table 1.

sub_class <- function(exp,geneid,pair_ne,pair_carci,pair_sclc){

sub_index <- function(exp,geneid,pair){

pairsid <- unique(c(pair[,1],pair[,2]))

newexp <- exp[which(geneid[,1] %in% pairsid),]

newgeneid <- geneid[which(geneid[,1] %in% pairsid),1]

pairs <- pair[which((pair[,1] %in% newgeneid) & (pair[,2] %in% newgeneid)),]

score_system <- function(exp,pairs,geneid){

len_sam <- ncol(exp)

len_pairs <- nrow(pairs)

score <- matrix(data = NA, nrow = len_sam,ncol = 1)

for(i in 1:len_sam){

s=0

for (j in 1:len_pairs){

if(exp[match(pairs[j,1],geneid),i] > exp[match(pairs[j,2],geneid),i])

s = s + 1

}

score[i,1] <- s

}

return(score)

}

score <- score_system(newexp,pairs,newgeneid)

level <- c()

level[which(score < (dim(pairs)[1]/2))] <- 0

level[which(score >= (dim(pairs)[1]/2))] <- 1

index1 <- which(level==1)

index2 <- which(level==0)

return(list(index1,index2))

}

ne_index <- sub_index(exp,geneid,pair_ne)[[1]]

nonne_index <- sub_index(exp,geneid,pair_ne)[[2]]

result <- c()

result[nonne_index] <- ‘non-NE’

carci_index <- ne_index[sub_index(exp[,ne_index],geneid,pair_carci)[[1]]]

noncarci_index <- ne_index[sub_index(exp[,ne_index],geneid,pair_carci)[[2]]]

result[carci_index] <- ‘CARCI’

sclc_index <- noncarci_index[sub_index(exp[,noncarci_index],geneid,pair_sclc)[[1]]]

lcnec_index <- noncarci_index[sub_index(exp[,noncarci_index],geneid,pair_sclc)[[2]]]

result[sclc_index] <- ‘SCLC’

result[lcnec_index] <- ‘LCNEC’

return(result)

}

**References**

Aerts, H.J., Velazquez, E.R., Leijenaar, R.T., Parmar, C., Grossmann, P., Carvalho, S., et al. (2014). Decoding tumour phenotype by noninvasive imaging using a quantitative radiomics approach. *Nat Commun* 5**,** 4006. doi: 10.1038/ncomms5006.

Asiedu, M.K., Thomas, C.F., Jr., Dong, J., Schulte, S.C., Khadka, P., Sun, Z., et al. (2018). Pathways Impacted by Genomic Alterations in Pulmonary Carcinoid Tumors. *Clin Cancer Res* 24(7)**,** 1691-1704. doi: 10.1158/1078-0432.CCR-17-0252.

Bild, A.H., Yao, G., Chang, J.T., Wang, Q., Potti, A., Chasse, D., et al. (2006). Oncogenic pathway signatures in human cancers as a guide to targeted therapies. *Nature* 439(7074)**,** 353-357. doi: 10.1038/nature04296.

Botling, J., Edlund, K., Lohr, M., Hellwig, B., Holmberg, L., Lambe, M., et al. (2013). Biomarker discovery in non-small cell lung cancer: integrating gene expression profiling, meta-analysis, and tissue microarray validation. *Clin Cancer Res* 19(1)**,** 194-204. doi: 10.1158/1078-0432.CCR-12-1139.

Der, S.D., Sykes, J., Pintilie, M., Zhu, C.Q., Strumpf, D., Liu, N., et al. (2014). Validation of a histology-independent prognostic gene signature for early-stage, non-small-cell lung cancer including stage IA patients. *J Thorac Oncol* 9(1)**,** 59-64. doi: 10.1097/JTO.0000000000000042.

Director's Challenge Consortium for the Molecular Classification of Lung, A., Shedden, K., Taylor, J.M., Enkemann, S.A., Tsao, M.S., Yeatman, T.J., et al. (2008). Gene expression-based survival prediction in lung adenocarcinoma: a multi-site, blinded validation study. *Nat Med* 14(8)**,** 822-827. doi: 10.1038/nm.1790.

Eustace, A., Mani, N., Span, P.N., Irlam, J.J., Taylor, J., Betts, G.N., et al. (2013). A 26-gene hypoxia signature predicts benefit from hypoxia-modifying therapy in laryngeal cancer but not bladder cancer. *Clin Cancer Res* 19(17)**,** 4879-4888. doi: 10.1158/1078-0432.CCR-13-0542.

Fernandez-Cuesta, L., Peifer, M., Lu, X., Sun, R., Ozretic, L., Seidal, D., et al. (2014). Frequent mutations in chromatin-remodelling genes in pulmonary carcinoids. *Nat Commun* 5**,** 3518. doi: 10.1038/ncomms4518.

Grossmann, P., Stringfield, O., El-Hachem, N., Bui, M.M., Rios Velazquez, E., Parmar, C., et al. (2017). Defining the biological basis of radiomic phenotypes in lung cancer. *Elife* 6. doi: 10.7554/eLife.23421.

Hight, S.K., Mootz, A., Kollipara, R.K., McMillan, E., Yenerall, P., Otaki, Y., et al. (2020). An in vivo functional genomics screen of nuclear receptors and their co-regulators identifies FOXA1 as an essential gene in lung tumorigenesis. *Neoplasia* 22(8)**,** 294-310. doi: 10.1016/j.neo.2020.04.005.

Jabs, V., Edlund, K., Konig, H., Grinberg, M., Madjar, K., Rahnenfuhrer, J., et al. (2017). Integrative analysis of genome-wide gene copy number changes and gene expression in non-small cell lung cancer. *PLoS One* 12(11)**,** e0187246. doi: 10.1371/journal.pone.0187246.

Jiang, L., Huang, J., Higgs, B.W., Hu, Z., Xiao, Z., Yao, X., et al. (2016). Genomic Landscape Survey Identifies SRSF1 as a Key Oncodriver in Small Cell Lung Cancer. *PLoS Genet* 12(4)**,** e1005895. doi: 10.1371/journal.pgen.1005895.

Karlsson, A., Brunnstrom, H., Micke, P., Veerla, S., Mattsson, J., La Fleur, L., et al. (2017). Gene Expression Profiling of Large Cell Lung Cancer Links Transcriptional Phenotypes to the New Histological WHO 2015 Classification. *J Thorac Oncol* 12(8)**,** 1257-1267. doi: 10.1016/j.jtho.2017.05.008.

Karlsson, A., Jonsson, M., Lauss, M., Brunnstrom, H., Jonsson, P., Borg, A., et al. (2014). Genome-wide DNA methylation analysis of lung carcinoma reveals one neuroendocrine and four adenocarcinoma epitypes associated with patient outcome. *Clin Cancer Res* 20(23)**,** 6127-6140. doi: 10.1158/1078-0432.CCR-14-1087.

Kim, N., Kim, H.K., Lee, K., Hong, Y., Cho, J.H., Choi, J.W., et al. (2020). Single-cell RNA sequencing demonstrates the molecular and cellular reprogramming of metastatic lung adenocarcinoma. *Nat Commun* 11(1)**,** 2285. doi: 10.1038/s41467-020-16164-1.

Laddha, S.V., da Silva, E.M., Robzyk, K., Untch, B.R., Ke, H., Rekhtman, N., et al. (2019). Integrative Genomic Characterization Identifies Molecular Subtypes of Lung Carcinoids. *Cancer Res* 79(17)**,** 4339-4347. doi: 10.1158/0008-5472.CAN-19-0214.

Lee, E.S., Son, D.S., Kim, S.H., Lee, J., Jo, J., Han, J., et al. (2008). Prediction of recurrence-free survival in postoperative non-small cell lung cancer patients by using an integrated model of clinical information and gene expression. *Clin Cancer Res* 14(22)**,** 7397-7404. doi: 10.1158/1078-0432.CCR-07-4937.

Lohr, M., Hellwig, B., Edlund, K., Mattsson, J.S., Botling, J., Schmidt, M., et al. (2015). Identification of sample annotation errors in gene expression datasets. *Arch Toxicol* 89(12)**,** 2265-2272. doi: 10.1007/s00204-015-1632-4.

Miranda, A., Hamilton, P.T., Zhang, A.W., Pattnaik, S., Becht, E., Mezheyeuski, A., et al. (2019). Cancer stemness, intratumoral heterogeneity, and immune response across cancers. *Proceedings of the National Academy of Sciences of the United States of America* 116(18)**,** 9020-9029. doi: 10.1073/pnas.1818210116.

Peifer, M., Fernandez-Cuesta, L., Sos, M.L., George, J., Seidel, D., Kasper, L.H., et al. (2012). Integrative genome analyses identify key somatic driver mutations of small-cell lung cancer. *Nat Genet* 44(10)**,** 1104-1110. doi: 10.1038/ng.2396.

Rousseaux, S., Debernardi, A., Jacquiau, B., Vitte, A.L., Vesin, A., Nagy-Mignotte, H., et al. (2013). Ectopic activation of germline and placental genes identifies aggressive metastasis-prone lung cancers. *Sci Transl Med* 5(186)**,** 186ra166. doi: 10.1126/scitranslmed.3005723.

Subramanian, A., Tamayo, P., Mootha, V.K., Mukherjee, S., Ebert, B.L., Gillette, M.A., et al. (2005). Gene set enrichment analysis: a knowledge-based approach for interpreting genome-wide expression profiles. *Proc Natl Acad Sci U S A* 102(43)**,** 15545-15550. doi: 10.1073/pnas.0506580102.

Whitfield, M.L., George, L.K., Grant, G.D., and Perou, C.M. (2006). Common markers of proliferation. *Nat Rev Cancer* 6(2)**,** 99-106. doi: 10.1038/nrc1802.

Wilkerson, M.D., Yin, X., Hoadley, K.A., Liu, Y., Hayward, M.C., Cabanski, C.R., et al. (2010). Lung squamous cell carcinoma mRNA expression subtypes are reproducible, clinically important, and correspond to normal cell types. *Clin Cancer Res* 16(19)**,** 4864-4875. doi: 10.1158/1078-0432.CCR-10-0199.

Wilkerson, M.D., Yin, X., Walter, V., Zhao, N., Cabanski, C.R., Hayward, M.C., et al. (2012). Differential pathogenesis of lung adenocarcinoma subtypes involving sequence mutations, copy number, chromosomal instability, and methylation. *PLoS One* 7(5)**,** e36530. doi: 10.1371/journal.pone.0036530.

Yamauchi, M., Yamaguchi, R., Nakata, A., Kohno, T., Nagasaki, M., Shimamura, T., et al. (2012). Epidermal growth factor receptor tyrosine kinase defines critical prognostic genes of stage I lung adenocarcinoma. *PLoS One* 7(9)**,** e43923. doi: 10.1371/journal.pone.0043923.

Yoshihara, K., Shahmoradgoli, M., Martinez, E., Vegesna, R., Kim, H., Torres-Garcia, W., et al. (2013). Inferring tumour purity and stromal and immune cell admixture from expression data. *Nat Commun* 4**,** 2612. doi: 10.1038/ncomms3612.

Zhu, C.Q., Ding, K., Strumpf, D., Weir, B.A., Meyerson, M., Pennell, N., et al. (2010). Prognostic and predictive gene signature for adjuvant chemotherapy in resected non-small-cell lung cancer. *J Clin Oncol* 28(29)**,** 4417-4424. doi: 10.1200/JCO.2009.26.4325.
